# Effects of prior knowledge on brain activation and functional connectivity during memory retrieval

**DOI:** 10.1038/s41598-023-40966-0

**Published:** 2023-08-22

**Authors:** Dingrong Guo, Haoyu Chen, Lingwei Wang, Jiongjiong Yang

**Affiliations:** School of Psychological and Cognitive Sciences, Beijing Key Laboratory of Behavior and Mental Health, Peking University, Beijing, 100871 China

**Keywords:** Long-term memory, Hippocampus, Cortex, Human behaviour

## Abstract

Previous studies have shown that the ventral medial prefrontal cortex (vmPFC) plays an important role in schema-related memory. However, there is an intensive debate to what extent the activation of subregions of the hippocampus is involved in retrieving schema-related memory. In addition, it is unclear how the functional connectivity (FC) between the vmPFC and the hippocampus, as well as the connectivity of the vmPFC with other regions, are modulated by prior knowledge (PK) during memory retrieval over time. To address these issues, participants learned paragraphs that described features of each unfamiliar word from familiar and unfamiliar categories (i.e., high and low PK conditions) 20 min, 1 day, and 1 week before the test. They then performed a recognition task to judge whether the sentences were old in the scanner. The results showed that the activation of the anterior-medial hippocampus (amHPC) cluster was stronger when the old sentences with high (vs. low) PK were correctly retrieved. The activation of the posterior hippocampus (pHPC) cluster, as well as the vmPFC, was stronger when the new sentences with high (vs. low) PK were correctly rejected (i.e., CR trials), whereas the cluster of anterior-lateral hippocampus (alHPC) showed the opposite. The FC of the vmPFC with the amHPC and perirhinal cortex/inferior temporal gyrus was stronger in the high (vs. low) PK condition, whereas the FC of the vmPFC with the alHPC, thalamus and frontal regions showed the opposite for the CR trials. This study highlighted that different brain networks, which were associated with the vmPFC, subregions of the hippocampus and cognitive control regions, were responsible for retrieving the information with high and low PK.

## Introduction

Many studies have suggested that information that involves prior knowledge (PK) or schema is more easily remembered than information that does not^1–3^. Assimilating new information into a pre-existing neocortical knowledge system is important for maintaining stable memory representations. The ventral medial prefrontal cortex (vmPFC) and the hippocampus have been identified as playing important roles in modulating memory performance that is related to PK^2,3^. A prevailing view is that the vmPFC supports encoding and retrieving of events congruent with PK. Likewise, the vmPFC inhibits hippocampal activation^2,4^ or acts as a cortical hub, interlinking memory representations in other brain regions, to replace the hippocampus and support a stable memory over time^4^. With consolidation, memory representations that are congruent with PK are quickly shifted to the neocortex and do not rely on the hippocampus^5,6^.

Consistent with this view, studies on memory encoding have found that cortical regions, including the vmPFC and the angular gyrus, are more strongly activated in relation to congruent (vs. incongruent) conditions^2,3,7–13^. The activation in the hippocampus^8,14–16^ decreases when the information is congruent with PK. In addition, studies of the functional connectivity (FC) consistently indicated that the vmPFC-cortical coupling increases, whereas that of the vmPFC-hippocampus decreases during encoding of PK congruent (vs. incongruent) information^8,11,13,17,18^.

However, to what extent the hippocampus is differentially involved in retrieval of memories related to PK is still debated^3^. Some studies found that activation of the hippocampus decreased in relation to PK^16,19–21^, whereas others showed the opposite^22,23^ or failed to find significant medial temporal lobe (MTL) activation related to PK^24–26^. Few studies have explored the connectivity between the vmPFC and the hippocampus during retrieval over time and the findings are inconsistent^11,19,23^. If the vmPFC inhibits hippocampal activation, the vmPFC-hippocampus coupling should decrease in the condition of high PK, as shown in encoding studies. Some studies have shown this pattern on both immediate and delayed retrieval^11^, whereas others do not^19^.

In recent years, the distinction between the anterior and posterior hippocampus has provided an intriguing way to understand the role of PK in memory retrieval^23,27^. The anterior hippocampus is more involved in processing gist or schema-related representation, whereas the posterior hippocampus is more involved in processing perceptually detailed, highly specific representations^28^. In a study of Guo and Yang^23^, the anterior hippocampus was more responsible for schema-related and gist-like retrieval, whereas the posterior hippocampus had the opposite pattern when participants retrieved object-location associations. In addition, there is a dissociation of the vmPFC coupling with the anterior and posterior hippocampus for retrieving schema-consistent or -inconsistent memories. Audrain and McAndrews^27^ also showed that the coupling of anterior hippocampus and vmPFC during rest was associated with coarse memory in the congruent condition after participants learned object—scene pairs. The dissociated roles of the anterior and posterior hippocampus in the effect of PK suggest that subregions of the hippocampus play different roles in retrieving PK-related information. On the other hand, behavioral studies have shown that PK also facilitates memory with details and perceptual features^17,29–32^, but whether the hippocampus is involved in this processing is unclear.

In addition to the vmPFC-hippocampus coupling, the connectivity of other regions with the vmPFC should be taken into consideration. Based on the schema theories^2,4,9,33^, because PK facilitates memory integration with pre-existing knowledge, vmPFC-cortical connectivity should be enhanced due to PK. However, only a few studies have explored the connectivity associated with cortical regions and the results were inconsistent^11,16,19,24,34^.

In contrast to memory encoding, memory retrieval includes various distinct processes^35^. During memory retrieval, a cue is first identified, and memory search is responsible for finding appropriate information based on the cue and the task demand^33^. Then inappropriate information or interference, such as similar information or lure options, must be inhibited or overcome by pattern separation and memory control^36,37^. Finally, when a choice is determined, the post-retrieval process plays its role to judge to what extent the choice is correct or appropriate. For example, compared to judging a stimulus as “old,” correctly rejecting a stimulus as “new” relies more on top-down control processes to detect novel signals, resolve interference and finally reject lures. So making a correct “new” response (i.e., CR) may induce more activation in the lateral prefrontal cortex^37–40^. Brod et al.^25^ showed that dorsolateral prefrontal cortex was more strongly activated when schema-incongruent information is retrieved. But so far, whether PK moderates vmPFC connectivity with memory control regions is unclear.

In addition, retention interval is an important factor to account for the effect of PK^5,6^. Studies have suggested that the effect of PK is more pronounced after longer intervals^20,27,41^. Sweegers et al.^19^ found that connectivity between the vmPFC/ACC and hippocampus increased from 30 min to 48 h when the information was related to PK. The retention interval was minutes after encoding in the study of Guo and Yang^23^. Both recent and remote retention intervals (especially longer than 24 h) should be included to clarify the functional connectivity in schema-related memory retrieval^6,11,13,19,27^.

In sum, the objective of the current study was to clarify to what extent the activation of subregions of the hippocampus, as well as FC between the vmPFC and brain regions including the hippocampus, were modulated by PK during memory retrieval over time. PK was defined as familiar category knowledge^8,29,42^. Sentences were used as stimuli to describe unfamiliar words, which were selected from familiar and unfamiliar categories (e.g., animal as a familiar category and bird as an unfamiliar category^30^). To explore whether the effect of PK on brain activation differed over time, three retention intervals (i.e., 20-min, 1-day, and 1-week) were included. Participants performed a recognition task to distinguish old from new sentences in the fMRI scanner. To dissociate the process of retrieving old events and rejecting interferences, the Hit and correct rejection (CR) trials were analyzed separately.

We hypothesized that both the vmPFC and the anterior hippocampus are involved in schema instantiation, thus they have significant PK effect during memory retrieval. The vmPFC also has significant FC with the subregions of the hippocampus to facilitate retrieving information with high and low PK, separately. In addition, because PK enhances detailed memory performance and recollection process^29,43–45^, the activation in the posterior hippocampus is stronger for high vs. low PK condition especially for the CR trials. In regard to cortical regions, the vmPFC would have stronger FC with control regions such as the lateral prefrontal cortex to facilitate retrieving information with low vs. high PK^25^ for the CR trials.

## Materials and methods

### Participants

Twenty-eight right-handed subjects (14 males) with a mean age of 21.23 ± 1.94 years participated in the study. All participants were native Chinese speakers. They provided written informed consent in accordance with the study approved by the Ethical Review Board of School of Psychological and Cognitive Sciences, Peking University. All methods were performed in accordance with the relevant guidelines and regulation.

### Materials

Two within-subjects factors were included in the study: level of PK (high and low) and retention interval (20-min, 1-day, and 1-week).

The materials were the same as Chen et al.^30^. We first selected 12 familiar (e.g., vegetable) and 12 unfamiliar categories (e.g., insect) based on Battig and Montague (1969) and van Overschelde et al.^46,47^. The familiarity of the 24 categories was also rated by 19 participants (13 males, with a mean age of 22.6 ± 2.58 years) who were not enrolled in the fMRI scanning and thus pre-determined before the experiment. For each category, the participants were asked to rate whether they were familiar with its general knowledge^29^ and whether they could generate many of its exemplars^46,47^ (l for most unfamiliar and 7 for most familiar). The mean scores for familiar and unfamiliar categories were 5.25 ± 0.77 and 3.99 ± 0.73, respectively. The difference was significant, *F* (1,18) = 256.71, *p* < 0.001, η^2^ = 0.93, which confirmed the validity of category selection. We then selected unfamiliar exemplars within each category and generated paragraphs for them. Each paragraph contained the name of the category the object belongs to, two sentences for perceptual features (e.g., color) and two sentences for functional features (e.g., usage)^48^. Each paragraph during encoding contained 36.36 ± 4.64 Chinese characters (including punctuation). Because an old/new recognition test was adopted during the retrieval phase, we also generated incorrect paragraphs for features with similar aspects (e.g., red vs. yellow in color) to be as foils at the time of tests.

Because the information in each paragraph is semantic-based general knowledge and may be acquired before the experiment, to reduce the influence of the confound, we controlled for the judgment accuracy for the sentences without learning at chance level (i.e., baseline accuracy). The correct and incorrect sentences were all presented separately and judged by another 18 participants (seven males, with a mean age of 21.0 ± 2.0 years) who did not learn the sentences in advance. The average accuracy (0.48 ± 0.03) was comparable to chance level (*p* > 0.30). There was no significant effect of PK (*p* = 0.26).

A total of 72 paragraphs were randomly divided into three sets. Half paragraphs described exemplars with high PK and the other half described exemplars with low PK. The three sets were used as materials for three retention intervals. Each paragraph was divided into four sentences by two perceptual and two functional features during retrieval (96 total for each interval), and the average length of the sentence was 9.27 ± 1.64 characters. The three sets had no significant differences in baseline accuracy, exemplar familiarity and sentence length (*ps* > 0.20). The sets were counterbalanced, and thus, each set had an equal chance of being used in different retention intervals.

### Procedure

**Figure 1:**
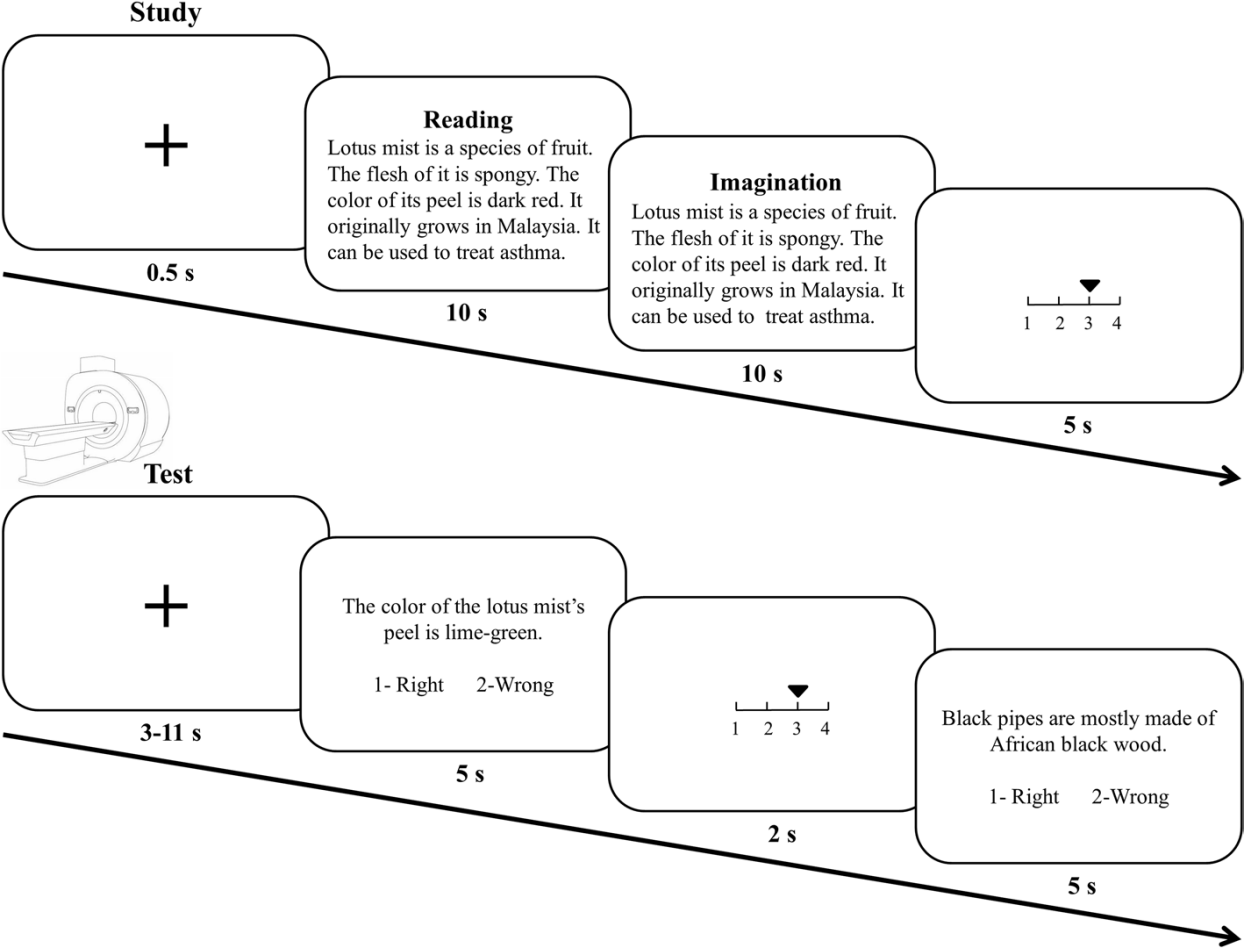
Procedure of the study and test phases. During the study phase, the participants were presented with paragraphs that described each unfamiliar exemplar from familiar and unfamiliar categories. During the test phase, the participants were asked to judge whether the sentence was correct followed by the confidence rating. The Chinese paragraphs are translated into English for illustration purpose.

The participants learned the paragraphs 20 min, 1 day, and 1 week before the test. They then performed the recognition test for all the paragraphs in the fMRI scanner 20 min after they learned the 20-min paragraphs (thus the interval was 20 min, 1 day and 1 week). During each study phase, the participants were randomly presented with each of the 24 paragraphs for 10 s with the instruction of “reading”, during which they read the whole paragraph silently (Fig. 1). The same paragraph was then presented for another 10 s with the instruction of “imagination”, during which they imagined a scene associated with the information that was described in the paragraph and judged the vividness (1–7, least to most). Within each paragraph, the category description was located at first, then the four sentences, with the order of the four sentences was counterbalanced across the participants.

During the test phase in the scanner, two sentences of each paragraph were presented as correct and the other two were as incorrect. As half sentences had high PK and the other had low PK, there were 24 sentences for each PK condition at each interval. The category sentences were not presented. Each of the sentences was presented for 5 s, and the participants were asked to judge whether the description was correct, followed by the confidence rating from unsure (1) to very sure (4). The sentences were pseudo-randomly presented at each retention interval for each participant so that no more than three sentences for each condition were presented consecutively. The correct/incorrect sentences and button press for the recognition judgment were counterbalanced across the participants. The perceptual and functional features in the correct and incorrect sentences were also counterbalanced across the participants. The inter-trial interval was adjusted in the event-related design to an average of 7 s (range: 3–11 s). The total of 288 trials were randomly divided into six runs, with each run having 48 trials and totaling 492 s (including the first six repetition times (TRs) for magnetic stabilization).

Before the test phase, the participants were asked to count backward by 7 continuously from 1000 for 5 min to avoid rehearsal. The participants had separate opportunities to practice study and test trials before the formal phases.

### MRI acquisition

The MRI data were collected on a Siemens Magneton Prisma 3 T scanner (Siemens Healthcare, Erlangen Germany) with a 20-channel head-neck coil. High-resolution functional images were acquired using a prototype simultaneous multi-slice echo-planar imaging (EPI) sequence (TR = 2000 ms, echo time [TE] = 30 ms, flip angle = 90, field-of-view [FOV] = 90°, number of slices = 64, matrix = 112 × 112, resolution = 2 × 2 × 2 mm^3^). This sequence applies a multiband pulse with slice-selective gradient to simultaneously excite multiple slice planes^49^. It has an advantage to reduce the scan time without reducing the signal-to noise rate or spatial resolution. Anatomical images were acquired using a high-resolution T1-weighted magnetization-prepared rapid gradient-echo (MP-RAGE) sequence (TR = 2530 ms, TE = 2.98 ms, flip angle = 7°, FOV = 22 cm, matrix = 256 × 256, resolution = 1 × 1 × 1.3 mm^3^) after the functional scanning.

### Image analysis

The AFNI software package was used for the fMRI analyses^50^. Using the afni_proc.py, the preprocessing script was generated. Briefly, the EPI volumes were linearly registered, smoothed with a 3D full-width at half-maximum (FWHM) of 6 mm, and scaled to a voxel-wise mean of 100. They were then warped into the Talairach and Tournoux^51^ atlas before the individual subject analysis (3dDeconvolve). The alignment was done by align_epi_anat.py in AFNI. This script computes the transforms needed to align EPI and anatomical datasets using a cost function designed for this purpose. The script combines multiple transformations, thereby minimizing the amount of interpolation applied to the data. In addition, the data was detrended with -polort 3 in the AFNI 3dDeconvolve, which is roughly equivalent to a high-pass filter with a cutoff of 1/D Hz, where 'D' is the duration of the imaging run.

During 3dDevonlve, estimates of brain activity related to each event for each participant were constructed via a general linear model. At each time interval, the events were defined as Hit, Miss, CR, and FA. Stimulus-evoked blood oxygenation level dependent (BOLD) responses to each event were modeled using AFNI's generalized additive models (GAM) response function adjusted for a 5-s stimulus duration. Because there were few FA and Miss trials, they were entered as variables of non-interest; Hit and CR events at each interval were included as variables of interest. Altogether, 12 regressors of interest (two levels of PK and three retention intervals by two trial types) and seven regressors of non-interest (six motion parameters and one non-interest variable) were applied, and the estimated β weights indicated the BOLD response amplitude for each condition. One participant was excluded because of excessive head motion during scanning (> 0.2 mm), and nine were excluded because of few Hit or CR trials (< 9 trials). In the end, data from 18 participants were entered into the fMRI data analysis.

To determine the difference between the experimental conditions, a voxel-wise mixed-effects ANOVA was performed with PK and retention interval as fixed-effects factors and subject as a random-effects factor. The group-level effects were identified on Hit and CR trials separately. The effects of PK and retention interval and their interactions were reported. In addition to cortical activation at the whole-brain level, we focused on the activation in the hippocampus and the vmPFC. Bilateral hippocampus masks were created using AFNI’s FS_Desai_PM atlas, which was originally parcellated by FreeSurfer^52^. The vmPFC anatomical mask was defined using the Mackey vmPFC Atlas^53^. To identify the typical effect of retrieval success (Hit vs. CR)^54,55^, a separate voxel-wise mixed-effects ANOVA was performed with trial type (Hit, CR), PK and retention interval as fixed-effects factors and subject as a random-effects factor (see Supplementary materials).

Furthermore, to explore whether the FC of the vmPFC with the hippocampus and other brain regions differed by PK and retention interval, the psychophysiological interaction (PPI)^56,57^ was applied. An independent vmPFC seed (9, 31, − 2)^23^ with a radius of 5 mm was first selected to identify the connectivity between the vmPFC and other brain regions. To perform the PPI analysis, all the regressors were convolved with the canonical hemodynamic response function using the AFNI's GAM response function adjusted for a 5-s stimulus duration^58^. The interaction regressors and the seed time-series regressor were then entered into the original univariate design matrix (i.e., 12 regressors of interest and seven regressors of non-interest). Finally, the β value associated with each interaction regressor was used in the ANOVA analysis for Hit and CR trials separately^57,59^.

The Monte Carlo simulation for the correction was performed with the AFNI program 3dFWHMx and 3dClustSim^60,61^. These versions incorporate a mixed autocorrelation function (ACF) that better models non-Gaussian noise structure^60,61^. The isotropic voxel size was 2 × 2 × 2 mm^3^ in our study. The activation and FC were determined at the level of voxel-wise *p* < 0.001 in combination with a minimum cluster extent of 67 voxels to maintain a family-wise error (FWE) rate of *p* < 0.05 in a whole-brain mask^62,63^. As we have specific predictions for the hippocampus and the vmPFC, the minimum cluster size for the corrected *p* of 0.05 (two-tailed) was determined in the hippocampus (small volume correction, SVC with 60 voxels) and in the vmPFC (SVC with 150 voxels) at the level of voxel-wise *p* < 0.05 for their activations and FC.

## Results

### Behavioral results

**Figure 2:**
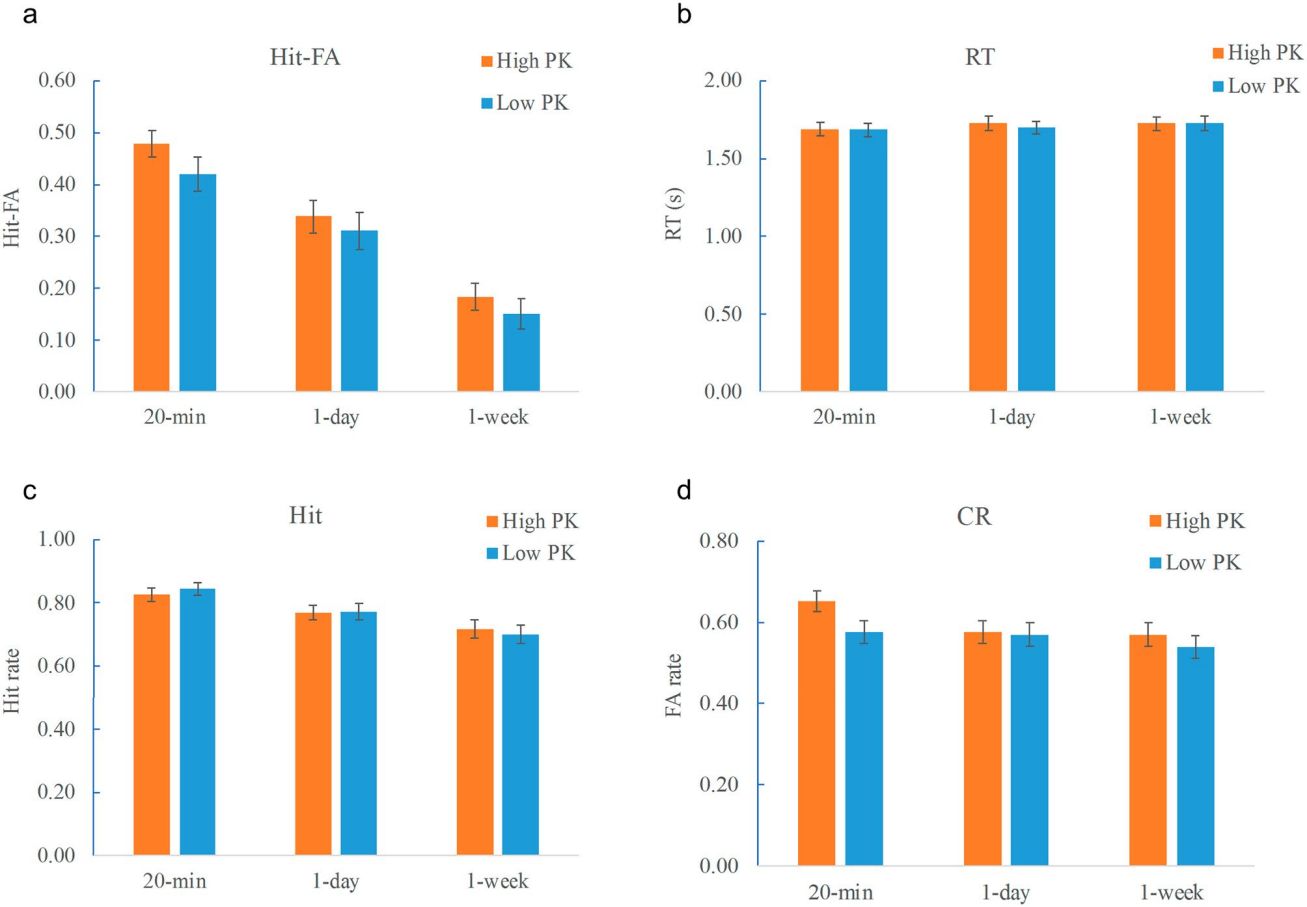
Behavioral results. (**a**) Corrected recognition. (**b**) RT. (**c**) Hit rate. (**d**) CR rate. The effect of PK was manifested in the corrected recognition and CR rate. There were no significant interactions between PK and interval for these parameters. Error bars represent the standard error of the mean (SEM).

The scores for the Hit rate, CR rate, corrected recognition (Hit-FA), and reaction times (RTs) were analyzed with a repeated-measures ANOVA with PK and retention interval as within-subjects factors. For the corrected recognition, the results showed that the sentences with high PK were recognized better than those with low PK (*F* (1,27) = 4.53, *p* = 0.04, η^2^ = 0.14) (Fig. 2a), and memory performance decreased significantly over time (*F* (2,54) = 49.64, *p* < 0.001, η^2^ = 0.65). No significant interaction between PK and interval was found (*F* (2,54) = 0.20, *p* = 0.82, η^2^ = 0.007). The RTs were comparable in different PK levels (*F* (1,27) = 0.97, *p* = 0.33, η^2^ = 0.04) (Fig. 2b), and increased over time (*F* (2,54) = 3.51, *p* = 0.04, η^2^ = 0.12). No significant interactions were found for the RTs (*F* (2,54) = 0.84, *p* = 0.44, η^2^ = 0.03).

The Hit rate decreased over time (*F* (2,54) = 20.61, *p* < 0.001, η^2^ = 0.43), but did not show a significant PK effect (*F* (1,27) = 0.009, *p* = 0.92), neither did the interaction between PK and retention interval (*F* (2,54) = 0.91, *p* = 0.41, η^2^ = 0.003) (Fig. 2c). The CR rate decreased over time (*F* (1,27) = 30.93, *p* < 0.001, η^2^ = 0.53), and a significant effect of PK was observed (*F* (1,27) = 8.71, *p* = 0.006, η^2^ = 0.24), showing that the sentences with high PK had higher CR rates (Fig. 2d). There was no significant interaction between PK and retention interval for the CR rate (*F* (2,54) = 1.74, *p* = 0.19, η^2^ = 0.06). The results confirmed the effect of PK over time with sentences as material, suggesting that PK provides a structure to enhance memory of detailed information through increasing the ability to distinguish targets from their lures.

The vividness rating during encoding and the confidence rating during testing were also analyzed. Sentences with high PK had higher vividness rating scores than those with low PK (*F* (1,27) = 7.72, *p* = 0.01, η^2^ = 0.23). The vividness was comparable during the two intervals (*F* (1,27) = 1.72, *p* = 0.19, η^2^ = 0.06), and no significant interaction between PK and retention interval was found (*F* (2,54) = 1.23, *p* = 0.30, η^2^ = 0.05). The confidence rating during retrieval decreased over time (*F* (1,27) = 95.12, *p* < 0.001, η^2^ = 0.78), with higher confidence rating in high than low PK condition (*F* (1,27) = 25.85, *p* < 0.001, η^2^ = 0.49). No significant interaction between PK and retention interval was found for the confidence rating (*F* (2,54) = 1.63, *p* = 0.20, η^2^ = 0.06).

### fMRI results

#### Hit trials

For the Hit trials, there was no significant effects of PK and retention interval, neither their interaction in cortical regions at the whole-brain level. For the SVC-corrected regions (i.e., hippocampus and vmPFC), because the hippocampal activity was distributed in separate clusters in this study, based on recent studies^64–66^, we functionally defined the hippocampus as three subregions, i.e., anterior-medial (amHPC), anterior-lateral (alHPC), and posterior (pHPC) hippocampus. The difference between our definition and that of Poppenk et al.^67^ was that the anterior hippocampus was further differentiated as the amHPC and alHPC subregions.

**Figure 3:**
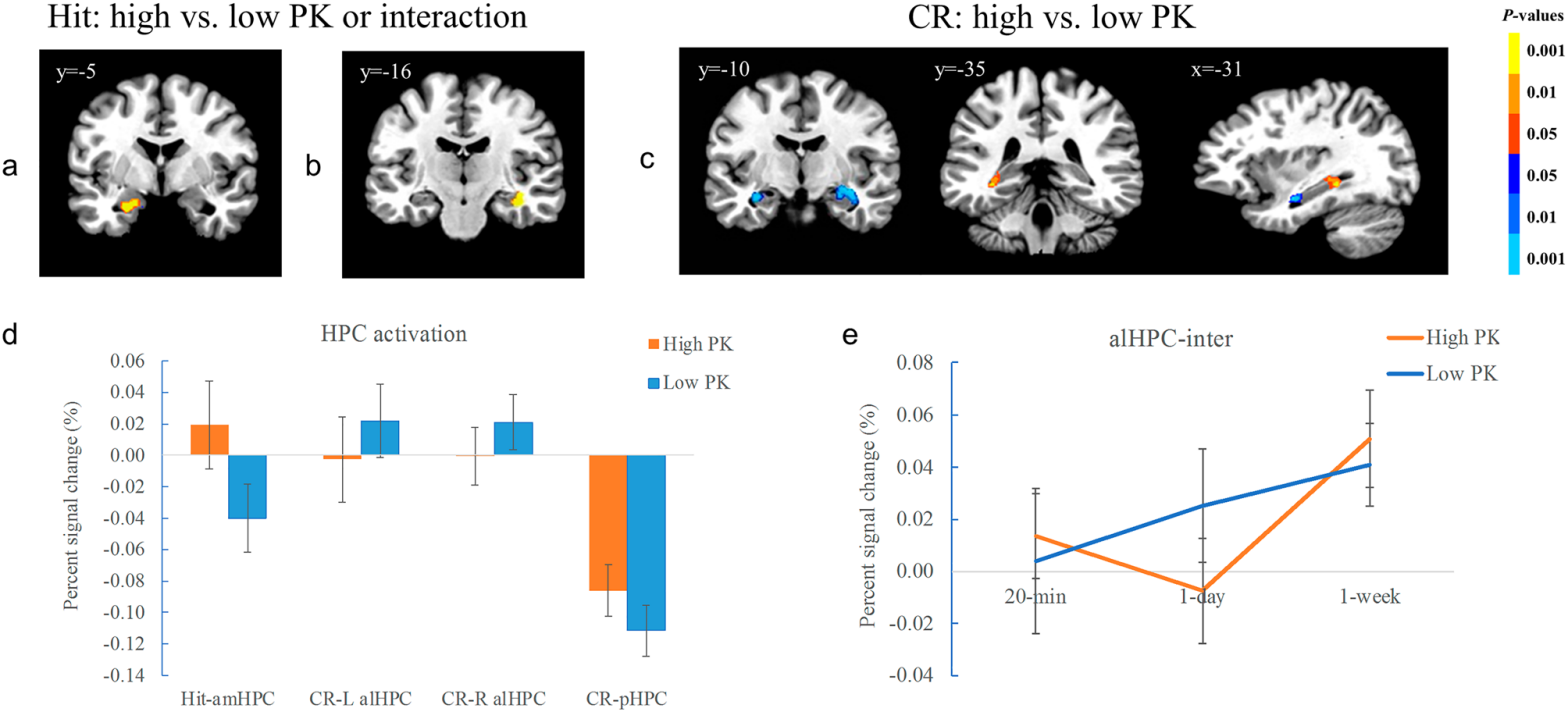
Voxel-wise results for Hit and CR trials in the hippocampus. (**a**) the cluster of the left amHPC showed significant effect of PK for the Hit trials. (**b**) The cluster of the right alHPC showed significant interaction between PK and interval for the Hit trials. (**c**) the clusters of the alHPC and pHPC showed different effect of PK for the CR trials. (**d**) plots showing signal changes of the hippocampal clusters in different PK condition for the Hit and CR trials. The data were collapsed across the retention interval. (**e**) plot showing signal change of the right alHPC cluster in each condition for the Hit trials. Color bars represent *p*-values, with the warm colors representing increased activation and the cold colors decreased activation for the contrast of high vs. low PK in A and C. The warm color represented interaction between PK and interval in D. The left is on the left side for each coronal brain slice. Error bars represent the SEM.

The results showed that there was a significant cluster in the left amHPC that was modulated by PK (− 23, − 5, − 20, *t*(17) = 4.18, *p* < 0.001) (Fig. 3a). The sentences with high PK elicited stronger activation in the amHPC (Fig. 3d), and this pattern did not change significantly over time (*p* > 0.05). In addition, there was a significant cluster in the right alHPC that had interaction between PK and retention interval (35, − 19, − 14, *F*(2, 34) = 20.49, *p* < 0.001) (Fig. 3b, 3e). Its activation was stronger for the sentences with low PK at the 1-day interval (37, − 17, − 14, *t*(17) = -4.93, *p* < 0.001), but did not show significant PK difference at other intervals (*p* > 0.05).

#### CR trials

For the CR trials, the ANOVA of PK condition by retention interval was also performed. The cortical regions showed distinct activation in the high vs. low PK conditions for the CR trials (Table 1) at the whole-brain level. The inferior frontal gyrus (IFG)/insula, anterior cingulate cortex (ACC), anterior temporal lobe (ATL), bilateral fusiform gyrus, and bilateral angular gyrus (AG) showed stronger activation in the low than in the high PK condition (*p* < 0.001). No significant activation was revealed for the effect of retention interval, neither the interaction between PK and interval (*ps* > 0.05). This suggests that compared to familiar sentences, rejecting incorrect unfamiliar sentences requires stronger activity in cortical regions that represent semantic knowledge and cognitive control processing for the CR trials.

**Table 1:** Voxel-wise results for the CR trials.

	Region	Peak	Voxels	*t* value	*p* value
High vs. low PK	Left IFG/insula	− 33, 17, 16	95	− 7.38	< 0.001
	Right ACC	19, 39, 8	120	− 5.68	< 0.001
	Left AG	− 33, − 57, 24	68	− 6.00	< 0.001
	Right AG	27, − 63, 30	104	− 6.29	< 0.001
	Left ATL	− 35, 7, − 20	115	− 7.60	< 0.001
	Left fusiform	− 25, − 47, − 20	333	− 7.02	< 0.001
	Right fusiform	39, − 63, − 6	77	− 6.06	< 0.001
	Right cerebellum	55, − 45, − 28	104	5.74	< 0.001
Interaction	N/A				
Time comparison	N/A				

**Figure 4:**
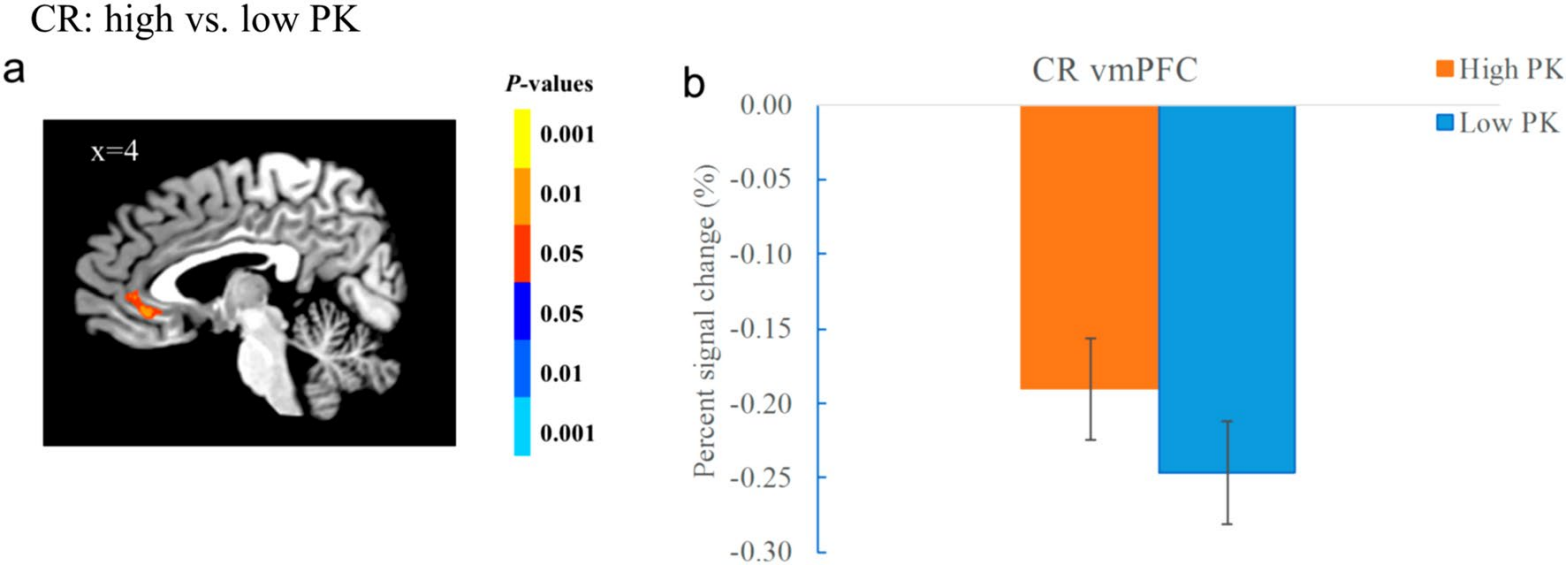
Voxel-wise results in the vmPFC for CR trials. (**a**) the vmPFC showed significant effect of PK for the CR trials. (**b**) plot showing signal change of the vmPFC in different PK condition for the CR trials. The data were collapsed across the retention interval in B. Color bars represent *p*-values, with the warm colors representing increased activation and the cold colors decreased activation for the contrast of high vs. low PK. Error bars represent the SEM.

For the SVC-corrected regions, the cluster in the bilateral alHPC (− 28, − 11, − 14, *t*(17) = -4.61, *p* < 0.001; 35, − 19, − 14, *t*(17) = -4.81, *p* < 0.001) showed stronger activation in the low than high PK condition, whereas that of the left pHPC (− 35, − 37, − 6, *t*(17) = 7.16, *p* < 0.001) showed the opposite (Fig. 3c, d). The vmPFC (5, 34, − 2, *t*(17) = 4.23, *p* < 0.001; 12, 34, − 12, *t*(17) = 5.09, *p* < 0.001) also showed stronger activation in the high than low PK condition (Fig. 4a, b). There was no significant effect of retention interval, neither the interaction between PK and interval (*ps* > 0.05), which suggests that these patterns do not change over time in the hippocampal clusters and the vmPFC for the CR trials.

### PPI results

**Figure 5:**
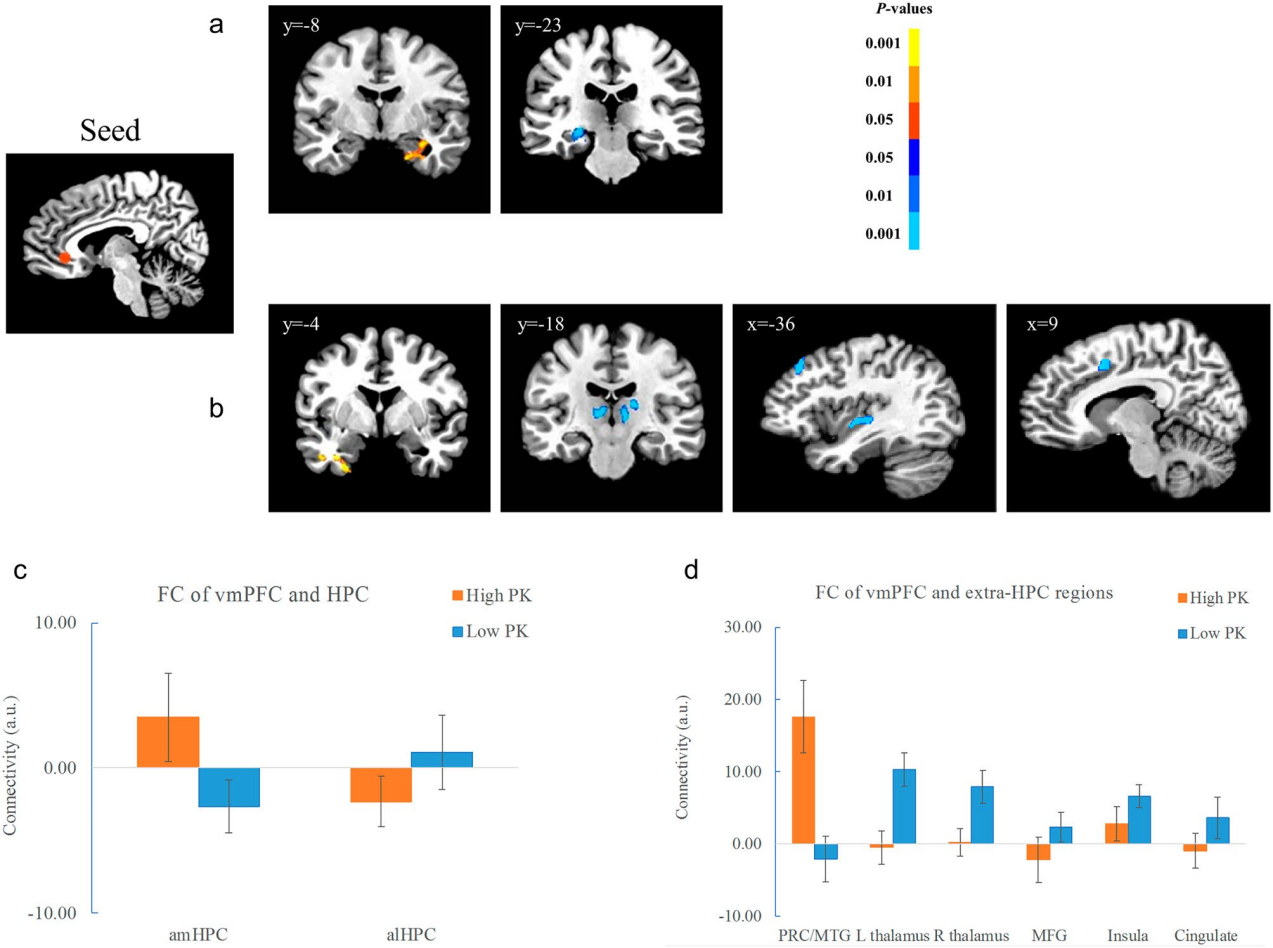
PPI results with the vmPFC as a seed. (**a**) The clusters in the amHPC and alHPC showed different effect of PK in the CR trials (right). The hippocampal activation is SVC-corrected. (**b**) The PRC/ITG showed stronger connectivity with the vmPFC for high vs. low PK condition, whereas the bilateral thalamus and cognitive control regions showed stronger connectivity with the vmPFC for low vs. high PK condition at the whole-brain level. (**c**) Plots showing the connectivity of the vmPFC with the hippocampal cluster in high and low PK conditions. (**d**) Plots showing the connectivity of the vmPFC with other regions in high and low PK conditions. The data were collapsed across the retention interval in C and D. Color bars represent *p*-values, with the warm colors representing increased activation and the cold colors decreased activation for the contrast of high vs. low PK. The left is on the left side for each coronal brain slice. Error bars represent the SEM.

To explore whether the FC between the vmPFC and brain regions differed by PK and retention interval, an independent seed of the vmPFC^23^ was selected (Fig. 5). As there were no significant effects or the interaction in brain regions for the Hit trials, only the results for the CR trials were reported. For the SVC-corrected regions, the results showed that the FC between the vmPFC and the cluster in the amHPC (33, − 7, − 14, *t*(17) = 3.51, *p* < 0.001) was significantly stronger in the high than low PK condition, whereas the FC between the vmPFC and the cluster in the alHPC (− 23, − 21, − 6, *t*(17) = 4.78, *p* < 0.001) was significantly stronger in the low than high PK condition (Fig. 5a, c). There was no significant effect of retention interval, neither the interaction between PK and interval (*ps* > 0.05).

At the whole-brain level, the FC of the vmPFC-PRC/inferior temporal gyrus (ITG) (− 23, − 7, − 34, *t*(17) = 6.80, *p* < 0.001) was significantly stronger in the high than low PK condition (Fig. 5b, d). In contrast, the FC of the vmPFC-thalamus (left: − 5, − 21, 6, *t*(17) = 6.66, *p* < 0.001; right: 17, − 19, 12, *t*(17) = 6.73, *p* < 0.001) was significantly stronger in the low than high PK condition (Fig. 5b, d). There was no significant effect of retention interval, neither the interaction between PK and interval (*ps* > 0.05). In addition, the FC between the vmPFC and the left insula (− 39, − 23, 2, *t*(17) = 6.33, *p* < 0.001), cingulate cortex (CC) (11, 7, 40, *t*(17) = 7.00, *p* < 0.001), left middle frontal lobe (MFG) (− 35, 29, 38, *t*(17) = 6.90, *p* < 0.001) (Fig. 5b, d), right middle temporal gyrus (MTG) (49, − 51, 6, *t*(17) = 4.97, *p* < 0.001), and precuneus (9, − 57, 52, *t*(17) = 6.74, *p* < 0.001) were stronger in the low than in the high PK condition.

## Discussion

The objective of this study was to explore to what extent the activation of the hippocampus, as well as the FC associated with the vmPFC was modulated by PK during memory retrieval over time. There were three main findings. First, the activation of the amHPC (for the Hit trials) and pHPC (for the CR trials) clusters was stronger when sentences with high (vs. low) PK were retrieved. The alHPC cluster showed the opposite pattern. Second, The FC of the vmPFC with the amHPC and alHPC clusters were dissociated in retrieving sentences in high and low PK conditions for the CR trials. Third, The FC of the vmPFC with PRC/ITG was stronger in the high (vs. low) PK condition, whereas the FC of the vmPFC with the thalamus, insula and prefrontal regions showed the opposite for the CR trials. These results highlighted that different brain networks, which were associated with the vmPFC and subregions of the hippocampus, as well as cognitive control regions, were responsible for retrieving the information with high and low PK.

### Long axis of the hippocampus and PK-related memory retrieval

Previous studies are inconsistent on to what extent the hippocampus is involved in retrieving information related to PK. A sentence recognition task in which the words were from either familiar or unfamiliar categories was used in this study. The materials were analogue to those were used in the classroom and our daily lives. The results showed that the amHPC and pHPC clusters elicited stronger activation related to PK, but their activation differed in retrieving old and rejecting new information. In contrast, the alHPC cluster showed stronger activation for retrieving sentences that were incongruent with PK. Moreover, the FC between subregions of the hippocampus and the vmPFC was functionally dissociated because of PK. The functional dissociation of hippocampal subregions and vmPFC—hippocampus connectivity appeared at shorter and longer intervals.

The results generally align with the view that subregions of the hippocampus play different roles in retrieving PK-related information. Previous studies have shown that the anterior and posterior hippocampus are separately associated with retrieving memory in regard to PK^23^. In addition, the anterior hippocampus and mPFC coupling was stronger for coarser memory as early as post-encoding^27^ and retrieval^27,68^. Cowan et al.^69^ showed that the hippocampus-vmPFC connectivity was modulated by the type of retrieved detail (i.e., object-word or scene-word associations). Consistently, we found that the amHPC cluster was more strongly activated when old sentences with high PK were correctly recognized, whereas the alHPC cluster showed the opposite, especially when new sentences with low PK were correctly rejected. Our study provided further evidence that the long axis of the hippocampus and their connectivity with the vmPFC is differentially involved in retrieving verbal information with high and low PK from 20-min to 1-week.

Although the functional distinction of anterior and posterior hippocampus has been used in recent years^67,70–72^, inconsistent findings are found especially for the anterior hippocampus^65,66,73,74^. For example, Thorp et al.^66^ showed that anterior–posterior parcellation may not sufficient to account for functional organization of the hippocampus. By data-driven approach, they found that the inter-voxel similarity (IVS) of the hippocampus is U-shaped, i.e., decreased along the medial–lateral axis of the anterior hippocampus but increased from anterior to posterior^66^. The results suggest that the hippocampal organization is complex and the anterior hippocampus could be differentiated as at least anterior-medial and anterior-lateral part. Different from previous studies that found anterior/posterior hippocampal distinction^23,27^, sentences were used as stimuli in this study. Whether the functional organization of the hippocampus is associated with materials needs further clarification.

The pHPC cluster showed stronger activation in the high vs. low PK condition for the CR trials. Our behavioral results showed that the CR rate was significantly higher in the high vs. low PK condition. Studies have suggested that when participants had to distinguish old and similar information, the ability to inhibit interference from lures is required^75,76^. The direct comparison of Hit vs. CR in this study (see Supplementary materials) showed that compared to the Hit trials, the CR trials elicited stronger activation in the bilateral PFC and SMG, as well as the ventral part of the vmPFC. The results confirmed that correctly rejecting new information require top-down processes and recollection contributions^33,37–39,77–80^. Similarly, the pHPC is identified to be associated with recollecting detailed information and inhibiting lures^81^, and having higher PK would facilitate the above processes. Behavioral studies have shown that information with PK not only enhances gist or conceptual-based memory, but also enhances detailed and perceptual-based memory^17,29–32^ and depends on recollection rather than familiarity process^43,44,82,83^. The pHPC is thus more involved in retrieving memory to help with the discrimination of old sentences from lure sentences in the high (vs. low) PK condition for the CR trials. This suggests that PK facilitates memory representations transferred from detailed/specific to coarser or more gist-like on the one hand, the nature of specific information influence the extent the hippocampus is involved in schema-related retrieval on the other hand^22,84^.

Therefore, although the participants remembered sentences associated with both familiar and unfamiliar categories at 1 week, the mechanisms may differ. Retrieving sentences with low PK is more associated with alHPC. In contrast, retrieving sentences with high PK is more associated with both the amHPC and pHPC.

### Connectivity of the vmPFC/hippocampus with other brain regions in PK-related memory retrieval

One novel finding of our study was that for the CR trials, the vmPFC-PRC/ITG coupling was stronger in the high vs. low PK condition, whereas the vmPFC-thalamus and vmPFC- lateral PFC coupling showed the opposite.

The PRC is regarded as a higher visual region that is associated with processing semantic knowledge. It is also involved in episodic retrieval of known statements and item information^85,86^. Other work shows that the mPFC is directly and bidirectionally connected to the PRC, lateral entorhinal cortex and MTG, which in turn is strongly connected bidirectionally with the anterior hippocampus^28,87^. In addition, the lateral temporal gyrus is important for storing content of semantic representation^88–90^. This region, including the ITG and MTG, has been involved in higher level of conceptual knowledge processing^90^. Our result of stronger coupling of the vmPFC and cortical regions in the higher PK condition was consistent with previous findings. For example, studies have shown that the connectivity of the vmPFC-precuneus/angular gyrus was enhanced in successful retrieval of the schema vs. non-schema map^11,24^. The vmPFC-fusiform gyrus coupling also increased for rule-based face-location memory at 48 h^19^. Thus, the coupling of the vmPFC—PRC/ITG is responsible for retrieving semantic representations that have been assimilated into the pre-existing knowledge system^91^.

In contrast, the vmPFC-thalamus coupling was stronger in the low vs. high PK condition. Previous studies have suggested that the thalamus is involved in recollecting memory representations and contextual memory encoding and retrieval^92–96^. Animal studies have shown that nucleus reuniens of the thalamus supports hippocampus-dependent encoding and retrieval of precise contextual memory^97,98^. Damage to the thalamus led to severe recall deficit in brain-lesioned patients^99–101^. Thalamic nuclei are in the center of the vmPFC-hippocampus connectivity^102,103^, mediating both hippocampal-vmPFC and vmPFC-posterior cortex connectivity during memory encoding, consolidation, and retrieval^87,94,96,104^. A study found that through the thalamic pathway, the vmPFC exerts top-down control of the hippocampus in retrieving context-appropriate memories and suppressing competing, context-inappropriate memories^87^. Our study further showed that the thalamus mediates the process that is more associated with low PK knowledge, especially for rejecting new information (in the CR trials).

In addition to the increased coupling of the vmPFC-thalamus in the low PK vs. high condition, our study showed that the couplings between the vmPFC and cognitive control regions such as the lateral PFC were also stronger in the low vs. high PK condition. The lateral prefrontal cortex is associated with semantic elaboration and integration^105^. In addition, the lateral prefrontal cortex, insula and the cingulate cortex are important for exerting processes for memory encoding and retrieval^37,106^. It seems that the schema-related network is different from the memory control network, although they are both involved in memory retrieval processes^25,68,107,108^. Brod et al.^25^ also found the vmPFC and lateral prefrontal cortex were dissociated in retrieving schema-congruent or -incongruent information. When the information has less PK, the brain regions responsible for semantic elaboration and memory control may work with the vmPFC to support memory retrieval.

Therefore, PK not only modulates the connectivity of vmPFC and subregions of the hippocampus, but also modulates the connectivity of vmPFC with other regions, including those are responsible for semantic processing, contextual and memory control processing. Different networks support successful memory retrieval whenever the information has stronger or weaker connections with PK.

### Stable memory representations and brain connectivity resulting from PK

In this study, to clarify whether the brain activation and connectivity resulting from PK changed over time, three retention intervals were included, i.e., 20-min, 1-day, and 1-week. But there were no significant interaction between PK and retention interval for most behavioral and fMRI results. One exception was that the activation in the alHPC cluster showed stronger activation in the low (vs. high) PK condition at the 1-day for the Hit trials, which was similar to that of the alHPC cluster for the CR trials.

The finding of stable memory representation and brain connectivity resulting from PK in this study was different from those in some previous studies. In general, retrieval purely based on schema would be faster and less effortful^25^. One difference between our finding and others^16,20,27^ is that the CR rate was significantly higher in the high versus low PK condition, but the RTs in the two conditions were comparable^85^. Our results suggest that the memory related to PK is enhanced through rejecting the interference more for schema-related information in our paradigm. As long as the memory representation retains its specificity, the correct recognition of old or new sentences that are associated with PK requires stronger hippocampus-vmPFC coupling, regardless of retention interval. The results supported the view that hippocampal activation is sustained when memory retrieval requires details^40,71,109–112^.

In addition, the PK-related sentences in our study were more semantic and were associated with pre-existing rather than training-based information, although they differ in familiarity. This may lead to fast consolidation, even right after encoding. Studies have suggested that consolidation is modulated by memory type (with item memory enhanced by PK after 20 h), but associative memory is enhanced right after the encoding^7^. It is suggested that PK or schema as the organizing scaffold serves to accelerate consolidation and neocortical integration of related memories^27,33,91^. If schema is manipulated as congruent to an existing or established knowledge system, memory representation could be rapidly formed to facilitate memory retrieval or predict incoming information.

Nevertheless, we are cautious to conclude that the effect of PK did not change over time. Although the interaction between PK and retention interval was not significant in our study, the RK difference between each interval varied in magnitude especially for the hippocampal activation and the connectivity between vmPFC and control system. Further study could enroll a larger sample of participants to replicate the findings.

### Memory retrieval related to PK over time

Combining the results of FC of vmPFC, our results showed that two distinct networks appeared to be associated with retrieving information related to PK. The network of the vmPFC, the amHPC, pHPC and the PRC/ITG was more responsible for retrieving information that had high (vs. low) PK, whereas the network of the vmPFC, the alHPC, thalamus and cognitive control regions was more responsible for retrieving information that had low (vs. high) PK. Among the two networks, the vmPFC may be located in a central position to coordinate the retrieval of information with high and low PK^2,3,91^. Consistent with the influential framework proposed by van Kesteren et al.^2^, the vmPFC is involved in assimilating a schematic or general representation into the pre-existing system and quickly consolidating them soon after encoding. At retrieval, schema instantiation directs a strategic memory search and provides a template for subsequent monitoring to ensure that the retrieved memory is consistent with the goals of the task^2,33,71^.

On the other hand, the model highlights that memory performance is a function of congruency, with better memory for incongruent information mediated by MTL^2^. The connectivity between the mPFC and MTL is also predicted to be inhibited during retrieval of congruent information. Different from the predictions, we found stronger activation of the amHPC cluster and stronger FC between the vmPFC and the amHPC cluster for high than low PK condition. It suggests that the dissociation of subregions of hippocampal function in PK-related memory retrieval should be considered. Particularly, the anterior part of the hippocampus is involved in memory retrieval by constructing a coherent scene or context^64,65^ especially when the existing knowledge system is available. Recent studies^23,27,68^, including the current study, thus extend the framework by differentiating the involvement of the hippocampus subregions and vmPFC-lateral PFC connectivity in PK-related processing. When a memory retrieval begins, the vmPFC, as a schema detector, is responsible for activating the appropriate context during schema reinstatement and instantiation through biasing posterior neocortical regions representing the exemplars related to appropriate PK^33,113,114^. At this stage, the coupling of the vmPFC-amHPC, and vmPFC-PRC/ITG may be more involved in retrieving gist-based information or the information with high PK^28,91^. At the same time, the stronger couplings of the vmPFC-alHPC, vmPFC-thalamus and vmPFC-cortical regions are more responsible for the retrieval of information with low PK or recovering perceptually representations of the event.

In contrast to the vmPFC networks, the contribution of the pHPC may work in a later stage of memory retrieval. Gurguryan et al.^115^ showed that the vmPFC—anterior hippocampus connectivity supports initial episodic construction of an autobiographical memory when it is first retrieved, and regions such as the pHPC support subsequent retrieval shortly thereafter. So it is possible that after a memory schema or representation has been constructed, the pHPC may contribute to memory retrieval through recollecting details and contextual information and inhibiting interference.

### Limitations

There are some limitations that can be addressed in future investigations. First, we found a significant effect of PK only on behavior for the CR trials. The behavioral features may influence the activation and FC results by emphasizing the processes such as detecting novel information and rejecting interference. Whether Hit-related memory retrieval resulting from PK is different from that of CR-related retrieval in the hippocampus and the related connectivity needs further clarification. Individual-based familiarity manipulation may be helpful to address this issue. Second, one limitation of fMRI technique is its relatively lower temporal resolution. Our study suggests that PK modulates both memory retrieval and memory control systems, but the assumption that the two mechanisms are coordinated in time scale needs to be tested in future studies. Third, in recent years, there are debates on whether the long axis of the hippocampus is functionally distinct or gradually changes^64,66,70^. In addition to the level of PK, other factors such as level of details and material type may also be associated with the hippocampal activation and connectivity in the hippocampal subregions. Future study could directly manipulate these factors to address this issue.

## Conclusions

In sum, the current study found that subregions of the hippocampus and their FC with the vmPFC showed different patterns in regard to PK. In addition, the coupling of the vmPFC-PRC/ITG and vmPFC-thalamus was stronger when new information was rejected in the high or low PK condition. This study highlighted that different brain networks, which were associated with the vmPFC and subregions of the hippocampus, were responsible for retrieving the information with high and low PK. PK also enhanced vmPFC-cortical FC, to exert top-down control to signal the need for interference resolution.

### Supplementary Information

Supplementary Information.

## Data Availability

The datasets used and analyzed during the current study are available from the corresponding author on reasonable request.
