# Supplementary material for: Effects of prior knowledge on brain activation and functional connectivity during memory retrieval
Source: Sci Rep. 2023 Aug 22;13:13650. doi: 10.1038/s41598-023-40966-0 (PMC10444832; doi:10.1038/s41598-023-40966-0)
Supplement: Supplementary file 1 — Supplementary Information. [file 41598_2023_40966_MOESM1_ESM.docx]

# Hit vs. CR

To explore the effect of retrieval success (Hit vs, CR), a voxel-wise mixed-effects ANOVA was performed with trial type (Hit, CR), PK and retention interval as fixed-effects factors and subject as a random-effects factor (*p* < 0.001). The results showed a typical old/new effect (i.e., Hit > CR) in the posterior medial regions (including PCC, precuneus, retrosplenial cortex), which was consistent with the conclusion by Wagner et al. (2005) ^[54]^. The anterior/middle cingulate cortex, mPFC and the cerebellum had the same pattern. The bilateral prefrontal cortex (PFC) and the supramarginal gyrus (SMG, left: -53, -59, 24; right: 47, -49, 24) showed the opposite pattern (i.e., Hit > CR) (Figure S1-a). In addition, there was a significant interaction between trial type and PK in the left PRC, left insula, left precentral gyrus and left MFG (Figure S1-b). These regions showed significantly stronger activation for Hit than CR trials only in the high PK condition (*ps* < 0.001 except the left MFG at *p* < 0.01 uncorrected). No other trial type-related interactions were found.

For the SVC-corrected regions of hippocampus and vmPFC, the results showed a positive old/new effect in the dorsal part of the vmPFC (-5, 37, 6, *t* (17) = 5.69, *p* < 0.001) and a negative old/new effect in the ventral part of the vmPFC (3, 23, -18, *t* (17) = -5.37, *p* < 0.001) (Figure S2-a). In addition, there was a significant trial type * PK interaction in the ventral part of the vmPFC (15, 39, -12, *F*(2, 34) = 23.94, *p* < 0.001) and the bilateral anterior hippocampus (left: -25, -5, -18, *F*(2, 34) = 21.79, *p* < 0.001; right: 33, -9, -12, *F*(2, 34) = 27.96, *p* < 0.001) (Figure S2-b). The bilateral anterior hippocampus showed significantly stronger activation for Hit than CR trials only in the high PK condition (*ps* < 0.001), whereas the ventral part of the vmPFC showed stronger activation for CR than Hit trials in the high PK condition (*p* < 0.001).

The results suggest that episodic memory retrieval is associated with two distinct networks, the default mode network (DMN) (including the midline regions and the hippocampus) is more involved in recognizing old items by maintaining representation of episodic or contextual details, whereas the executive and attentional network is more
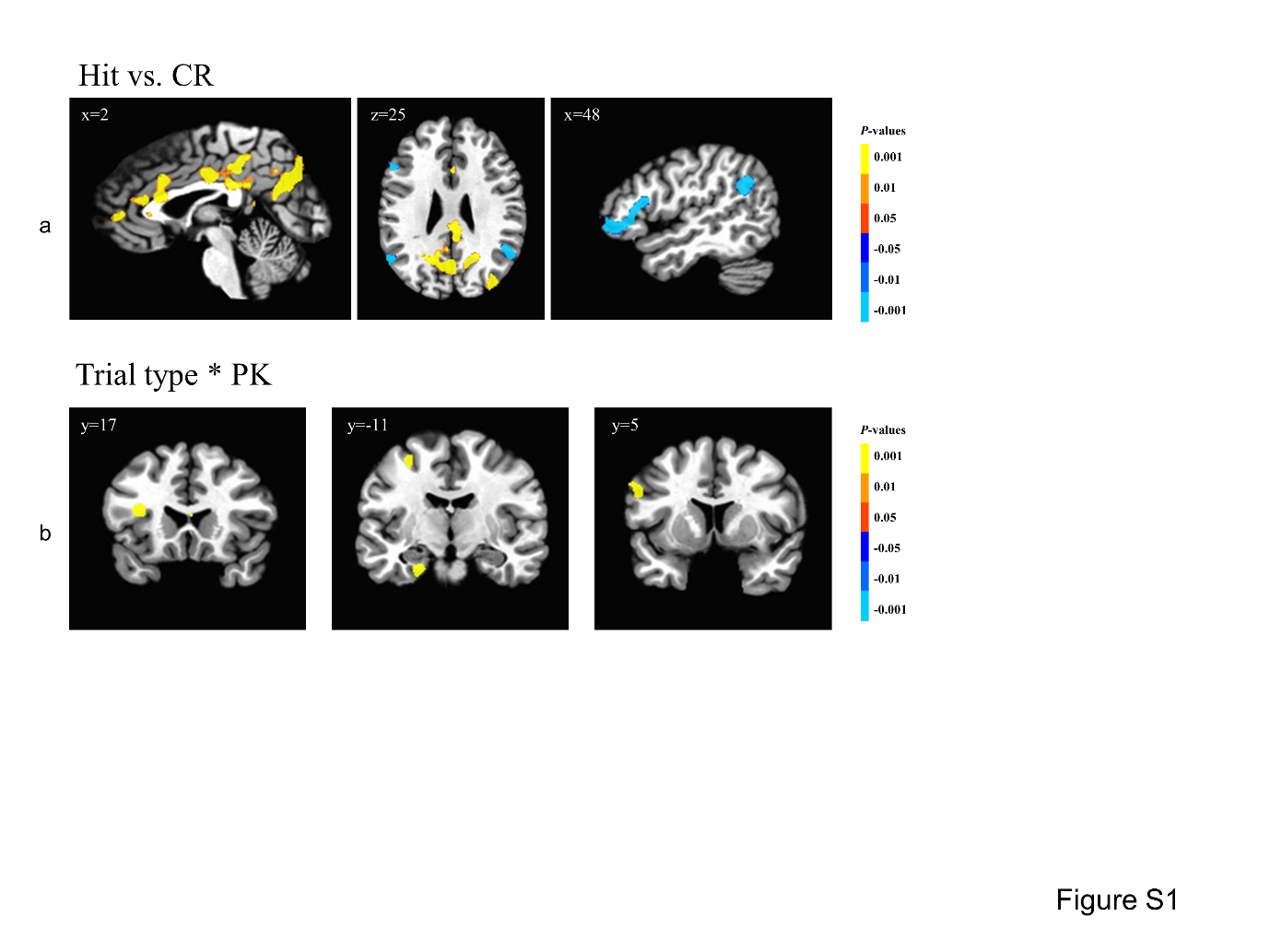
involved in rejecting new items.

Figure S1. Voxel-wise results for Hit vs. CR and the interaction of trial type and PK condition at the whole-brain level. Color bars represent *p*-values for the contrast of Hit vs. CR or the interaction of trial type * PK. The left is on the left side for each coronal or axial brain slice.
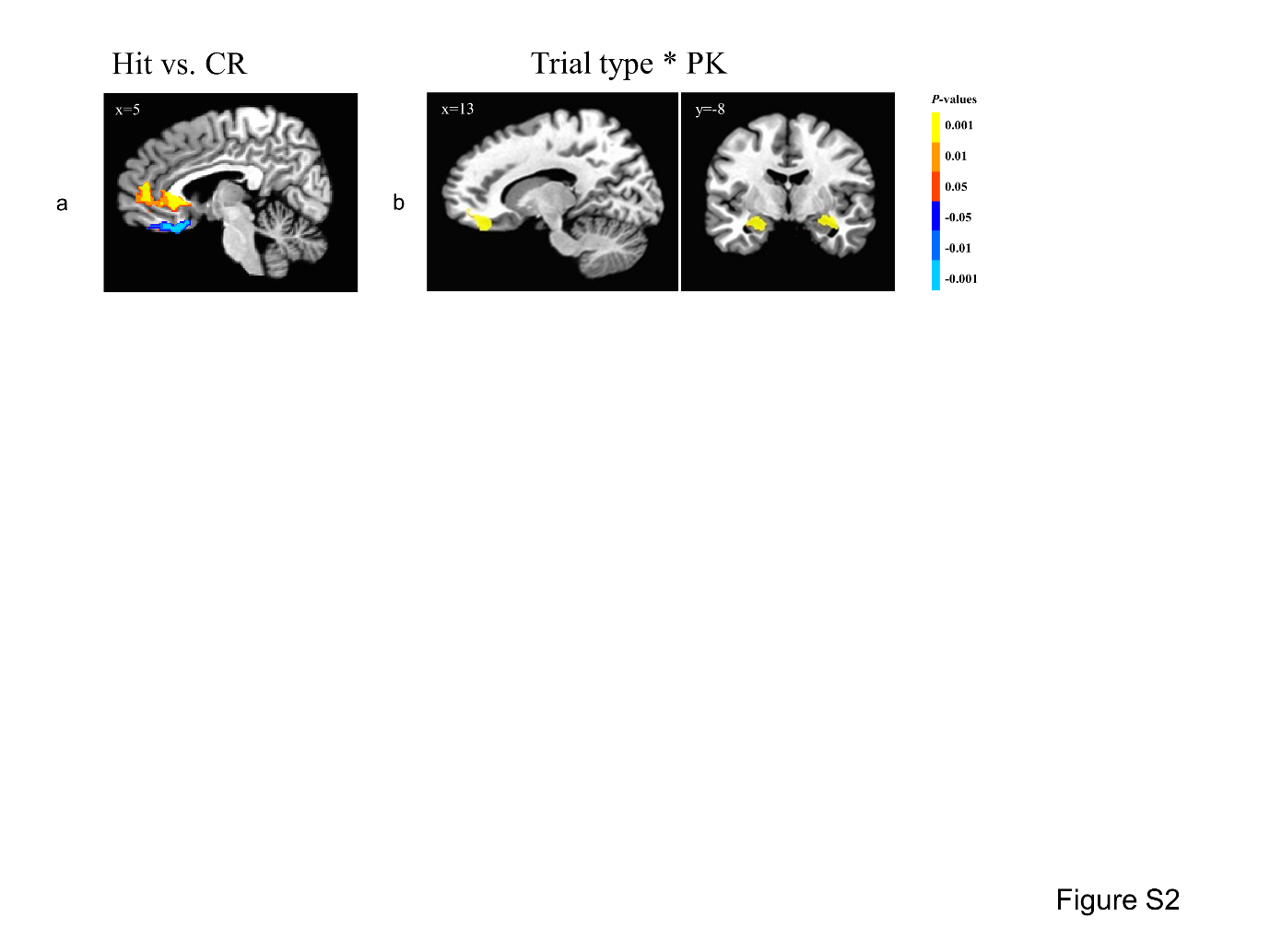


Figure S2. Voxel-wise results for Hit vs. CR and the interaction of trial type and PK condition in the SVC-corrected hippocampus and vmPFC. Color bars represent *p*-values for the contrast of Hit vs. CR or the interaction of trial type * PK . The left is on the left side for the coronal brain slice.
